# Identification of differentially abundant micro-organisms associated with Sjögren’s syndrome: a 16S rRNA sequencing data mining approach

**DOI:** 10.1093/femsle/fnag094

**Published:** 2026-08-18

**Authors:** Laura Losada Calderón, Sergio Andres Castañeda Garzon

**Affiliations:** Digital Health Laboratory, Faculty of Medicine, Universidad El Bosque, Bogotá, Colombia; Digital Health Laboratory, Faculty of Medicine, Universidad El Bosque, Bogotá, Colombia

**Keywords:** Sjögren’s syndrome, data mining, oral microbiota, microbial diversity, microbiome, metabarcoding

## Abstract

Manifestations of Sjögren’s syndrome (SS) considerably affect the quality of life. Owing to the multifactorial nature of the syndrome, some individuals present alterations in oral microbiota profiles. This study analyzed the oral cavity microbiota profiles of 242 samples—168 from pSS group and 74 from control group—using metabarcoding technique based on sequencing of 16S rRNA gene. Data processing, amplicon sequence variants, and taxonomic assignment were performed using DADA2. Statistical and microbial diversity analyses were performed using phyloseq in R. Alpha and beta diversity metrics were evaluated, as well as the identification of enriched taxa using Linear discriminant analysis effect size analysis. Results revealed no significant differences in the composition and structure of the microbiota between groups. However, differential abundance analysis allowed the identification of 27 microbial taxa, including species *Actinomyces dentocariosa*; genera *Streptococcus, Leptotrichia, Veillonella, Fusobacterium*, and *Alloprevotella*; and phyla Actinobacteriota and Fusobacteriota, in pSS group. Although no direct associations have yet been established between oral microbiota and SS, some of the identified genera have been documented to possess pathogenic factors that induce immune responses. Accordingly, this study lays the groundwork for future analyses of oral microbiota profiles in SS to identify potential microbial biomarkers of disease.

## Teaser

Discover how specific oral microbes, identified through an innovative data-mining approach, could be the missing link in understanding and treating Sjögren’s Syndrome.

## Introduction

Primary Sjögren’s syndrome (pSS) is a multisystemic autoimmune disorder that alters the mechanisms of autoimmunity, causing inflammation of the exocrine glands and loss of secretory function (Qin et al. 2015, Fernández-Ávila et al. 2020). It is mainly manifested as xerophthalmia and xerostomia, that is, ocular and oral dryness, respectively (Moreno-Useche et al. 2021, Reyes Jaraba et al. 2022). The syndrome is classified as primary when it occurs in isolation and secondary when it is associated with other autoimmune diseases, such as rheumatoid arthritis, Hashimoto’s thyroiditis, or systemic lupus erythematosus (Ruiz Gutiérrez et al. 2017, Moreno-Useche et al. 2021). The association with other autoimmune diseases, known as polyautoimmunity, implies that its diagnosis, treatment, and follow-up must be conducted systematically (Sarmiento-Monroy and Gómez-Puerta 2020, Vega Castro and Pertuz Charris 2020). SS is one of the most prevalent autoimmune disorders worldwide (Fernández-Ávila et al. 2020). Some studies estimate the prevalence of SS to range from 0.1% to 4.8% (Nagi et al. 2025), predominantly affecting women between 40 and 60 years of age (Fernández-Ávila et al. 2020, Reyes Jaraba et al. 2022).

The pathogenesis of SS remains incompletely understood and is considered multifactorial, involving genetic, immunological, hormonal, and external factors that induce an autoimmune response (Ruiz Gutiérrez et al. 2017, Sarmiento-Monroy and Gómez-Puerta 2020, Schaefer et al. 2022 , Zeng et al. 2022, Furlan Freguia et al. 2023). Among these, alterations in the oral microbiome have emerged as potential contributors to disease development, with recent studies suggesting that dysbiosis may influence disease onset and progression (Gupta et al. 2017, Alemán-Ávila et al. 2018, Moreno-Useche et al. 2021, Bustos-Lobato et al. 2023, Yang et al. 2023).

The oral microbiome plays an essential role in maintaining oral homeostasis by preventing pathogen colonization and modulating immune responses (Zorba et al. 2020, Sedghi et al. 2021). In pSS, oral microbiome dysbiosis has been associated with immune dysregulation, chronic inflammation, and salivary gland dysfunction (Deng et al. 2022, Bustos-Lobato et al. 2023). In addition to the oral cavity, the syndrome commonly presents with dry eye syndrome resulting from lacrimal gland dysfunction, and emerging evidence suggests that microbial communities at different mucosal sites, including the ocular surface, may also contribute to disease pathophysiology (Maślińska et al. 2021, Chang et al. 2022, Schaefer et al. 2022).

Accordingly, several studies support the relationship between microbiome alterations and the development of SS. Changes in the composition of the oral microbiome have been associated with greater symptom severity and immune dysfunction, whereas viral and bacterial infections have been proposed as important external factors influencing disease manifestation and autoantibody production (Maślińska et al. 2021, Deng et al. 2022, Chang et al. 2022). Given the close anatomical and functional relationship between the oral cavity and the salivary glands, numerous studies have focused on characterizing oral microbiome alterations associated with the pathological mechanisms of the disease (De Paiva et al. 2016, Bustos-Lobato et al. 2023). These alterations may contribute to salivary gland dysfunction, leading to hyposalivation and the characteristic symptom of xerostomia, and have been associated with changes in the relative abundance of genera such as *Streptococcus, Veillonella, Neisseria*, and *Porphyromonas* (De Luca and Shoenfeld 2019, Rusthen et al. 2019, Chang et al. 2022, Kim et al. 2022, Nagi et al. 2025).

Although some microbial taxa have been associated with pSS, variations in reported oral microbiome profiles across studies, likely reflecting population heterogeneity and methodological differences, underscore the need for integrated analyses of publicly available data. Therefore, the present study aims to characterize oral microbiota profiles in patients with SS using publicly available 16S rRNA sequencing data and to identify differentially abundant micro-organisms that may guide future studies focused on the identification of potential microbial biomarkers of this disease. This approach seeks to provide evidence that contributes to the understanding of the disease’s behaviour and its possible interaction with the pathophysiological mechanisms of the syndrome, and offers a study model applicable to other autoimmune diseases in which interactions with microbial factors play a relevant role.

## Materials and methods

### Study design and sample selection

This was a descriptive cross-sectional study with a non-probabilistic convenience sampling approach comprising two groups of oral cavity microbiota sequencing data: patients diagnosed with pSS (pSS group) and healthy individuals (Control group, not diagnosed with SS). The samples were collected through a search for research articles with open-access data via the PubMed database and a search equation based on standardized terms ((*Sjögren syndrome*) *AND* (*oral microbiota*)) *NOT* (*review*).

A total of 394 samples were obtained from public repositories, of which 245 met the selection criteria and were subjected to data processing. The selection criteria considered in this study included retrospective studies from 2016 to 2024 involving adult subjects with autoimmune diseases, specifically pSS. In addition, studies focused on describing the microbial communities of the oral cavity in pSS and control groups using Amplicon-Based Sequencing, generating 250 bp paired-end reads on Illumina MiSeq and HiSeq platforms and targeting the V3–V4 hypervariable regions of the 16S rRNA gene with the universal primers 341F and 806R. The studies included a description of the data obtained and ensured the availability of these data in public database repositories. The exclusion criteria included single-end sequencing data not compatible with the data processing pipelines, samples other than saliva, and patients without a confirmed diagnosis of pSS.

This study consisted of a secondary analysis of publicly available 16S rRNA sequencing data and did not involve the recruitment of participants or the collection of new biological samples. All original studies included in the analysis reported approval by their respective institutional ethics committees and written informed consent from all participants. In addition, the present study was approved by the Research Ethics Committee of El Bosque University (Approval No. 005–2025; Project code CIE-2025–015).

### Data preparation

All the data were downloaded in fastq format from public repositories and prepared prior to analysis. Paired-end reads were separated using the SRAToolkit [National Center for Biotechnology Information (NCBI) 2024] to obtain the forward and reverse reads for each sample, which are required for independent quality control and error correction processes. The study metadata were subsequently generated, containing the information on the samples and their classification according to the group to which they belonged (pSS—Control).

### Microbial diversity analysis

A quality profile assessment was performed using DADA2 (Callahan et al. 2015) in R to select samples with a Phred score greater than 30 and minimize erroneous reads. The samples were subsequently filtered, and the estimation of error rates was carried out to improve the resolution of the inference of amplicon sequence variants (ASVs). Next, the dereplication of identical sequences was performed to group them into unique sequences. A denoising process was then applied, employing a probabilistic model to identify and correct sequencing errors in the forward and reverse reads. Similarly, the merge pairs process was performed to combine the forward and reverse sequences of each sample based on the overlapping regions and base agreement, enabling the reconstruction of validated, high-accuracy sequences. Afterwards, the ASVs were determined, and a sequence table was constructed. Finally, chimera removal and taxonomic assignment were performed based on sequences from the Silva v138 reference database (Quast et al. 2013).

The diversity analysis was performed using the Phyloseq package (McMurdie and Holmes 2013) in R. A phyloseq object was initially constructed, integrating the DADA2 outputs containing the ASV abundance table, taxonomic assignment, and metadata. A filtering process was also applied to remove samples and taxa with unidentified ASV information. This enabled the calculation of Observed, Shannon, and Simpson diversity indices, relative abundance, identification of the most abundant phyla and genera in each analyzed group, and the construction of their corresponding plots. Additionally, linear discriminant analysis (LDA) effect size (LEfSe) (Segata et al. 2011) was performed to identify the differentially abundant taxa in each of the pSS and Control groups.

### Statistical analysis

Normalization of proportions based on scaled relative abundance was performed to correct for differences in sequencing depth across samples. Calculations and estimates of relative abundance and alpha diversity metrics, including Observed, Shannon, and Simpson diversity indices, were used to compare the pSS and Control groups. For the comparison of diversity indices between study groups, the non-parametric Mann‒Whitney‒Wilcoxon test was applied. Similarly, to evaluate beta diversity, the Bray‒Curtis dissimilarity metric and distance matrix were calculated, which allowed the generation of a Non-Metric Multidimensional Scaling (NMDS) ordination plot to visualize group behaviour. Permutational Multivariate Analysis of Variance (PERMANOVA) was conducted to assess whether the microbial composition differed significantly between the groups. Finally, a dispersion test was applied to evaluate the variability of samples within each group to determine whether differences were influenced by sample variability or reflected the microbial composition between groups. All tests were conducted using R software (R Core Team 2024), and *P values* < 0.05 were considered statistically significant.

## Results

### Sample results

A total of 245 samples from related previous studies were collected through a search of publicly available 16S rRNA paired-end sequencing data downloaded from the National Center for Biotechnology Information (NCBI) repositories and the Genome Sequence Archive (GSA) of the National Genomics Data Center (Table 1). A total of 7 398 228 reads were obtained from all the samples, with an average of 30 571 reads per sample, allowing the identification of 90 188 ASVs. After the samples containing low read counts and unidentified ASV information were filtered, the total number of samples was reduced to 242 with adequate quality for analysis, of which 168 samples belonged to the pSS group and 74 samples belonged to the Control group.

**Table 1:** Selected studies with public metagenomic data used for the construction of the pSS and Control groups.

Study	Reference	Accesion ID	Samples	Repository
Hyposalivation but not Sjögren’s syndrome associated with microbial dysbiosis in women	(Saúco et al. 2023)	PRJNA983278	pSS 25 Control 25	NCBI
Microbiota dysbiosis in primary Sjögren’s syndrome and the ameliorative effect of hydroxychloroquine	(Wang et al. 2022)	PRJCA008752	pSS 129 Control 36	GSA
Salivary dysbiosis in Sjögren’s syndrome and a commensal-mediated immunomodulatory effect of salivary gland epithelial cells	(Tseng et al. 2021)	PRJNA693659, PRJNA693663	pSS 14 Control 16	NCBI
Source: Authors.				

### Alpha diversity analysis

**Figure 1:**
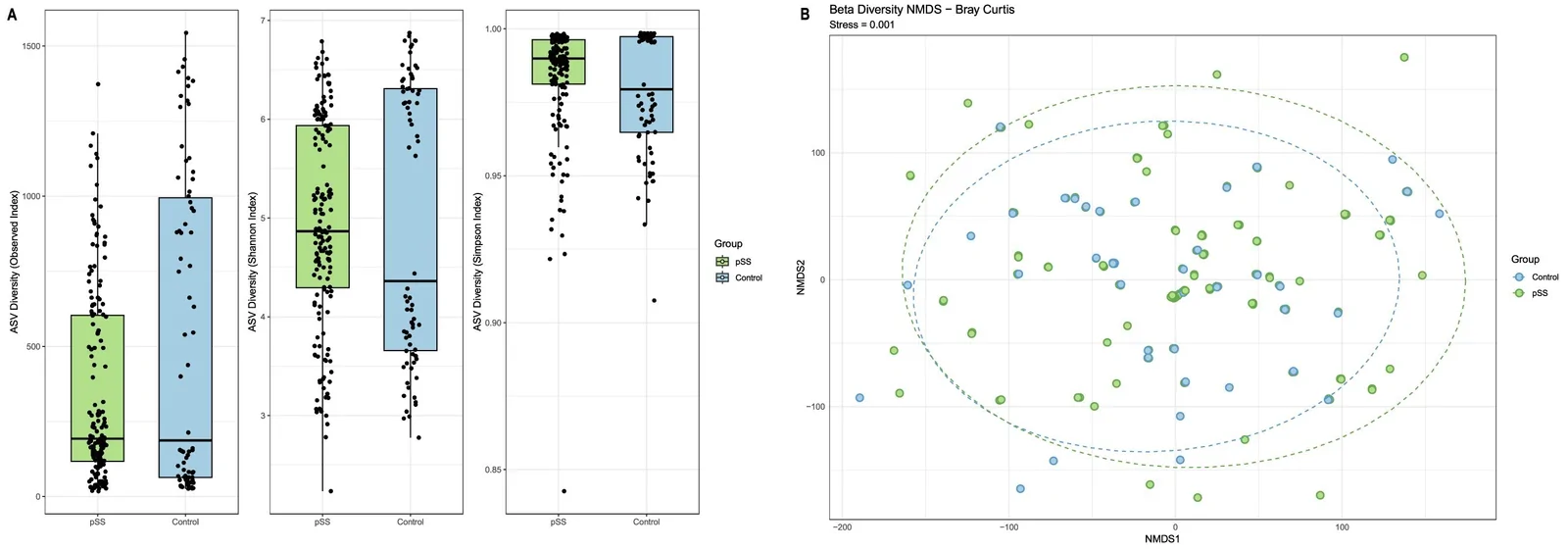
Alpha and beta diversity analysis between SS patients and Control groups. (A) Alpha diversity metrics—Observed, Shannon, and Simpson indices—by pSS and Control sample groups. (B) NMDS ordination based on Bray–Curtis dissimilarity of the microbial communities from pSS and Control groups.

Alpha diversity analysis was performed using the Observed, Shannon, and Simpson diversity indices for the pSS and Control groups, and no statistically significant differences were detected (Fig. 1A). Similarly, the pSS and Control groups were compared using the non-parametric Mann‒Whitney‒Wilcoxon test for the Observed (*P* = 0.3268), Shannon (*P* = 0.8319), and Simpson (*P* = 0.904) indices, confirming the absence of statistically significant differences in the alpha diversity of the experimental conditions.

### Beta diversity analysis

Similarly, beta diversity analysis was performed using NMDS, which adequately represented the multivariate structure of the data under real conditions, as indicated by the *Stress* value (*Stress* = 0.001) (Fig. 1B). PERMANOVA based on the Bray‒Curtis dissimilarity metric was performed, yielding a significant value that was not consistent with the NMDS plot (adonis2 *P* = 0.001, *R²* = 0.00595). A dispersion test was then performed to evaluate sample variability (betadisper *P* = 0.001), which revealed a significant difference in the dispersion of the groups. Despite the statistical test being significant, no clear compositional difference was observed between the study groups, indicating that the result may be driven by heterogeneity in within-group variance.

### Relative abundance analysis

**Figure 2:**
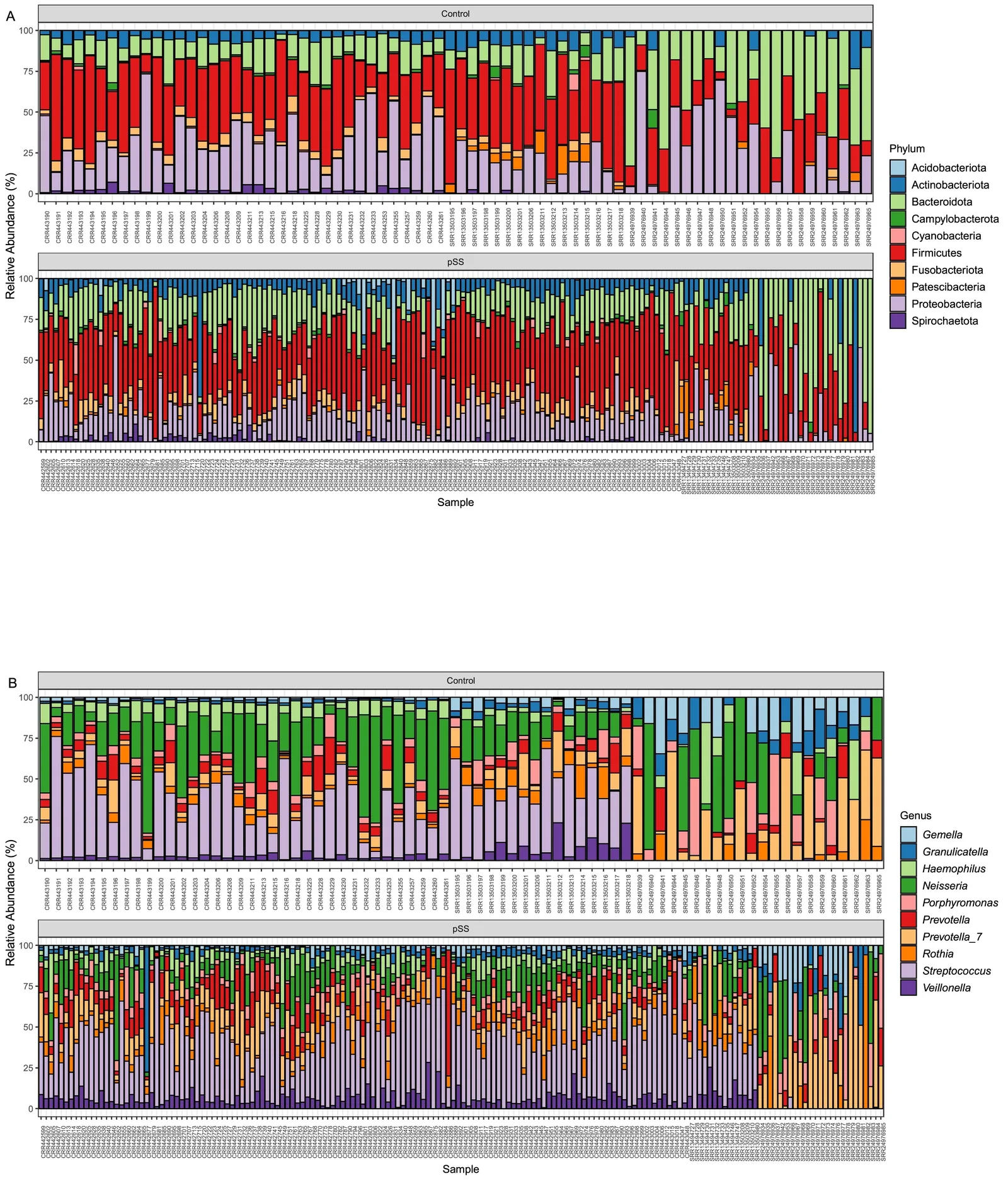
Bar charts of top relative abundance between SS patients and Control groups. (A) Relative abundance bar chart at the phylum level. (B) Relative abundance bar chart at the genus level.

To characterize the oral microbiota of patients with SS and healthy individuals, the top 10 most abundant phyla and genera in the group samples were identified (Fig. 2). The oral microbiota of both the pSS and Control groups was dominated by a similar core composition. At the phylum level, Firmicutes, Proteobacteria, and Bacteroidota, along with Actinobacteriota, presented the highest abundances in the pSS group (Fig. 2A). Similarly, at the genus level, the pSS and control groups presented greater abundances of *Streptococcus, Neisseria*, and *Prevotella*; however, differences were observed in less abundant taxa, with *Veillonella* and *Granulicatella* more frequently detected in pSS samples than in control samples, whereas *Gemella* was consistently present in controls (Fig. 2B). The genus level analysis also revealed a difference in the samples from the study conducted by Saúco et al. in Spain, in which *Streptococcus* and *Veillonella* were not represented within the top 10 in relative abundance; however, this does not indicate their absence in these samples and may be related to population variations within the analyzed dataset.

### Differential abundance analysis

**Figure 3:**
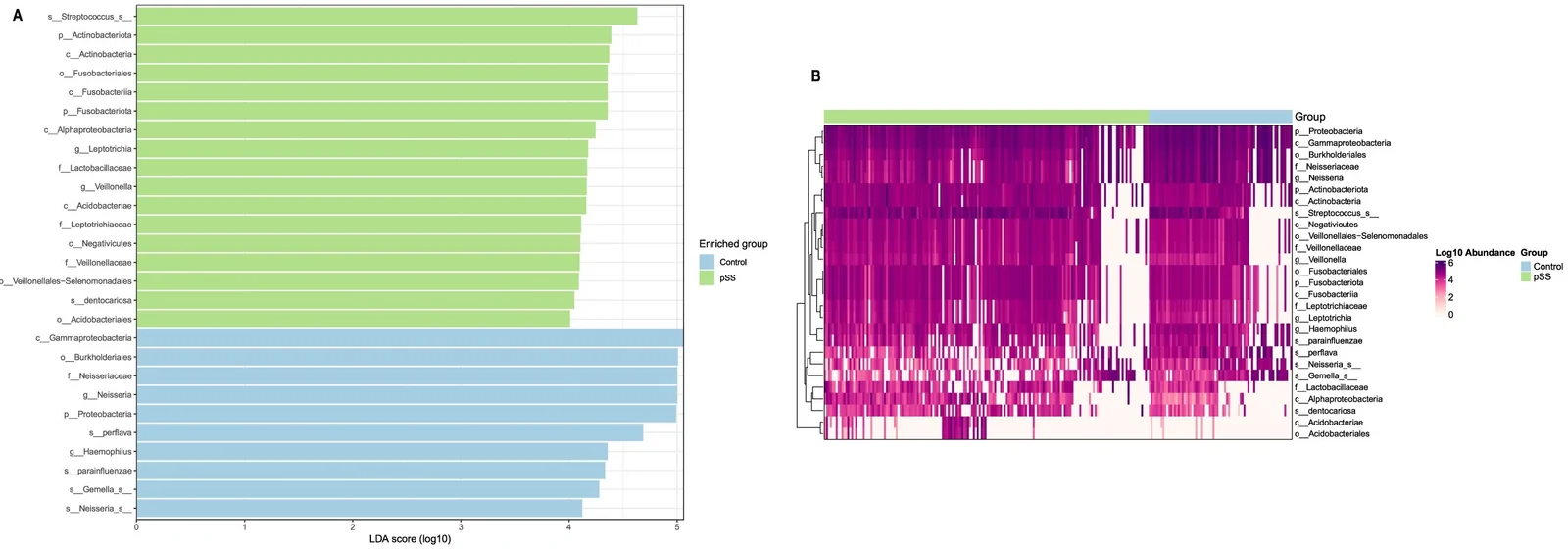
Differential abundance analysis. (A) Bar plot showing enriched taxa across different taxonomic levels in pSS and Control groups. (B) Heatmap of differentially abundant taxa between pSS and Control groups.

To identify the taxa significantly more abundant in the pSS and Control sample groups, LEfSe was applied using an LDA score threshold of 4. This analysis revealed differentially enriched taxa ranked according to the magnitude of their LDA score differences (Fig. 3A), identifying a total of 27 microbial taxa. In the pSS group, significant increases in the abundances of the phyla Actinobacteriota and Fusobacteriota; the genera *Streptococcus, Leptotrichia, Veillonella, Fusobacterium*, and *Alloprevotella*; and the species *Actinomyces dentocariosa* were detected, which is consistent with the findings from the relative abundance analysis. Conversely, in the Control group, significant increases were detected in the abundances of the phylum Proteobacteria; the genera *Gemella, Neisseria*, and *Haemophilus*; and the species *Neisseria perflava* and *Haemophilus parainfluenzae*. Similarly, the differences described are represented in a heatmap based on relative abundances (Fig. 3B), allowing a comparative visualization of the distributions of the main taxa identified between the pSS and Control groups.

## Discussion

Recently, alterations in the oral microbiota have been considered risk factors for the development of autoimmune diseases, as certain commensal micro-organisms can become opportunistic pathogens due to their virulence factors and ability to evade the immune response. Nevertheless, the pathogenesis of these diseases remains unclear, and questions persist regarding the factors involved in their early stages (Huang et al. 2023, Yuan et al. 2025).

In this context, several studies have suggested that patients with SS exhibit a distinct oral microbiota profile compared with healthy individuals (Rusthen et al. 2019, Bustos-Lobato et al. 2023, Huang et al. 2023, Kamounah et al. 2025). Given that the oral cavity is anatomically accessible and easily sampled, compositional analyses of the oral microbiome have enabled the identification of micro-organisms across different sites and the comparison of their community structures (Huang et al. 2023). From this perspective, our study focused on characterizing the oral microbiota in patients with SS and identifying differentially abundant micro-organisms associated with this disease.

Alpha diversity analysis revealed no significant differences in the microbiota between patients with SS and healthy individuals (Fig. 1A). Recent studies have reported similar findings, without identifying a specific microbial profile characteristic of SS (Tseng et al. 2021, Saúco et al. 2023, Xie et al. 2024). These results may be attributed to uncontrolled experimental conditions and methodological factors, such as the small sample size—fewer than 35 participants per group—and analyses limited to the genus level, among others, which may restrict the detection of relevant outcomes (Tseng et al. 2021, Saúco et al. 2023).

In the beta diversity analysis, compositional heterogeneity of the microbiota was observed between patients with SS and healthy individuals (Fig. 1B). The study conducted by Bustos-Lobatoet al. attributed this heterogeneity to variations in salivary secretion volume and concentration (Bustos-Lobato et al. 2023), which could explain the intragroup variability observed in this and other studies (Tseng et al. 2021, Wang et al. 2022, Saúco et al. 2023). The composition of oral microbial communities varies significantly across different niches—saliva, mucosa, tongue, teeth, or gums—due to local conditions characterized by specific pH, temperature, hydration, and immunological factors that modulate microbial growth (Bustos-Lobato et al. 2023). Within this framework, the reduction in salivary microbial composition may be related to hyposalivation, a characteristic symptom of the syndrome (Siddiqui et al. 2016, Chahal et al. 2022, Chang et al. 2022, Wang et al. 2022, Bustos-Lobato et al. 2023, Saúco et al. 2023).

In the relative abundance analysis, the oral cavity was taxonomically characterized (Fig. 2), describing prevalent genera such as *Streptococcus, Neisseria*, and *Prevotella* (Saúco et al. 2023) and phyla such as Firmicutes, Proteobacteria, and Bacteroidota (Tseng et al. 2021, Wang et al. 2022), which is consistent with studies showing that the structure and composition of the oral microbiota in patients with SS are similar to those observed in healthy individuals (Tseng et al. 2021, Saúco et al. 2023, Xie et al. 2024). The species belonging to the described genera are characterized as commensal bacteria of the oral cavity that, under certain conditions, can behave as opportunistic pathogens and contribute to inflammatory processes and disease development, as well as altering glandular functions. In line with these results, Xie et al. identified *Prevotella melaninogenica* as a species related to increased interferon production and dysregulation of the submandibular gland, suggesting the involvement of specific taxa in the pathophysiology of the disease (Xie et al. 2024).

However, in this relative abundance analysis, differences were observed in the sample group from the study conducted by Saúco et al., which could be attributed to the population heterogeneity of our study groups, as these samples correspond to a Spanish population, in contrast to the other analyzed cohorts originating from China and Taiwan. Cohort heterogeneity may encompass a set of geographic differences associated with genetic, environmental, and cultural factors that may influence the composition of the microbiota (Gupta et al. 2017). In addition, variations in sample collection procedures, participant management, and sequencing methodologies may further contribute to the observed variability.

The differential abundance analysis identified dominant taxa in the groups (Fig. 3), yielding findings that are consistent with scientific evidence (Kim et al. 2022, Xie et al. 2024, Tseng et al. 2025). First, the oral microbiota in healthy individuals was characterized by predominant genera such as *Haemophilus* and *Neisseria*, and by the phylum Proteobacteria (Xie et al. 2024). Studies by Kim et al. and Tseng et al. reported a significant decrease in these genera in patients with SS compared with healthy individuals. In particular, species belonging to the genus *Haemophilus* exhibit immunomodulatory properties and contribute to maintaining host homeostasis (Kim et al. 2022, Tseng et al. 2025), participating in the regulation of microbial balance and immunity in both the salivary glands and the oral microbiota (Kim et al. 2022, Tseng et al. 2025). In agreement with these findings, this study observed an enrichment of *Haemophilus spp*., particularly *Haemophilus parainfluenzae*, in the control group, suggesting that *Haemophilus spp*. is a characteristic component of a balanced oral microbiota (Tseng et al. 2025).

In general, a healthy microbiota contributes to host health by maintaining balance and appropriately regulating defence mechanisms. However, disturbances in this equilibrium may induce dysregulated responses that promote the development of immune-mediated diseases. Given this, it is particularly relevant to analyse the organisms enriched in patients with SS, as their presence may constitute a key factor in the disease’s pathophysiology (Tseng et al. 2025).

In this regard, the oral microbiota of patients with pSS was characterized by the predominance of genera such as *Veillonella* and *Streptococcus*, as well as phyla such as Actinobacteriota and Fusobacteriota (Xie et al. 2024). These taxa may play a relevant role in the manifestation of the disease by participating in key microbial interactions, particularly those modulated by pathogenic factors. Specifically, *Veillonella* species have been described as anaerobic bacteria with immunomodulatory properties and identified in the oral microbiota of patients with SS (Nagi et al. 2025). These species, considered bridging organisms, interact directly or indirectly with pathogenic species, promoting their colonization in the oral cavity (Zhou et al. 2021, Xie et al. 2024, Yuan et al. 2025).

Likewise, species of the genus *Streptococcus*, although considered commensal micro-organisms, may adopt opportunistic behaviours when the mucosal barrier is altered and contribute to the development of various pathologies. Consistent with this, Kim et al. reported a correlation between oral microbiota dysbiosis and the enrichment of this genus in the analyzed samples (Kim et al. 2022). In particular, *Streptococcus mutans* and *Streptococcus oralis* stand out for their active participation in the early formation of dental biofilms and in pathological processes such as caries and periodontitis. These species also have the ability to trigger systemic infections under specific conditions, thereby enhancing microbial imbalance (Zorba et al. 2020, Sedghi et al. 2021, Li et al. 2022, Chahal et al. 2022, Ren et al. ). Consequently, the proliferation of these commensal species with pathogenic potential promotes microbial disequilibrium that increases host susceptibility to bacterial infections, which is consistent with the higher prevalence of caries, oral candidiasis, and periodontitis frequently documented in patients with SS (Huang et al. 2023, Zhou and Liu 2023, Yuan et al. 2025).

During the development of these conditions, the colonization of micro-organisms with pathogenic potential in the salivary glands disrupts local homeostasis, induces inflammation, and promotes epithelial cell dysregulation. The affected cells become targets for T lymphocytes, contributing to increased apoptosis and secretory dysfunction that characterize the clinical manifestations of the syndrome. In parallel, these processes trigger biological mechanisms that induce an immune response mediated by the infiltration of B and T lymphocytes into the salivary glands (Alam et al. 2020, Chang et al. 2022, Deng et al. 2022). According to Huang et al., the biological mechanisms involved include (i) molecular mimicry, in which structural similarities between microbial peptides and autoantigens may induce a cross-reactive autoimmune response; (ii) microbial translocation, which allows micro-organisms to reach other tissues, generate systemic inflammation, and affect distant organs; (iii) overproduction of autoantibodies, secondary to pathogen-induced inflammation; and (iv) amplification of autoimmunity by cytokines, which is induced by micro-organisms that stimulate the release of proinflammatory cytokines (Deng et al. 2022, Huang et al. 2023).

In addition to biological mechanisms, Zeng et al. and Talotta, Sarzi-Puttini and Atzeni demonstrated that epigenetic mechanisms are involved in pathogen-mediated changes in cellular activity, influencing the expression of genes associated with the immune response. The mechanisms include: (i) DNA methylation, in which pathogens alter methylation patterns to activate or silence immune-related genes; (ii) histone modifications, which reshape chromatin structure and, consequently, the expression of inflammation-related genes; and (iii) microRNA regulation, which can be manipulated by pathogens to promote their survival or the transformation of infected cells (Talotta et al. 2019, Zeng et al. 2022).

Taken together, the evidence suggests that the pathophysiology of SS involves cellular interactions affected by organisms present in the oral cavity, reinforcing the role of the microbiota and bacterial infections in its development (Alam et al. 2020, Chang et al. 2022, Deng et al. 2022).

This study lays the groundwork for future analyses of the oral cavity microbiota profiles of patients diagnosed with autoimmune diseases, particularly SS, which could provide significant insights into its pathogenesis. Among its strengths, this study highlights the rigorous use of state-of-the-art bioinformatic tools, ensuring technically robust processing with single-nucleotide resolution and high taxonomic identification accuracy using the DADA2, Phyloseq, and LEfSe pipelines. Furthermore, it incorporated a broad dataset comprising samples from diverse international cohorts reported in studies by Saúco et al. (2023), Tseng et al. (2021), and Wang et al. (2022). The data-mining approach allowed for an in-depth exploration of the behaviour of the samples and groups analyzed, as well as the identification of differentially abundant microbial taxa in each group through a reproducible and scalable model.

Nevertheless, this study was constrained by several limitations. First, the sequencing depth of the samples was not homogeneous. Although the average number of reads per sample was adequate, some samples presented considerably low sequencing depth (< 5000 reads), which restricts the characterization of low-abundance taxa and may introduce biases in diversity analyses. Second, the high intragroup variability generated substantial internal dispersion, hindering the detection of true differences between groups and the identification of disease-associated taxa. Third, population heterogeneity across the different cohorts included in this study may have introduced additional variability in the observed group differences, which could affect the interpretation of the results. Furthermore, clinical variables directly related to the manifestations of SS were not considered, nor was metadata from the samples or sociodemographic information from the patients available, which limited the possibility of performing a complementary descriptive statistical analysis. Finally, the study underscores the need to integrate multiomics approaches—including genomics, transcriptomics, and epigenetics—together with clinical variables to strengthen the role of the microbiome in future studies aimed at identifying potential microbial biomarkers in SS through interactions with pathophysiological mechanisms.

## Conclusions

This study characterized the oral microbiota of patients with pSS in comparison with healthy individuals, identifying the microbial profiles and differentially abundant taxa of the disease. Although no statistically significant differences were observed in the microbial composition between groups, it was possible to determine the most abundant genera and phyla in each group, thus characterizing their respective microbial profiles. Similarly, enriched taxa were identified for each group and corroborated by previously reported scientific evidence.

These findings provide an effective strategy for microbiome analysis in autoimmune diseases, such as SS, based on sequencing and bioinformatics techniques. Furthermore, they lay the groundwork for a broader understanding of the disease’s behaviour and its possible interaction with genetic, immunological, and external factors, considering the relationships among different microbiota and the pathophysiological mechanisms involved. Finally, this study provides a reproducible and scalable methodological framework for future research aimed at understanding the potential interactions between microbial factors and the pathophysiological mechanisms of SS and other autoimmune diseases in which microbial factors may play a relevant role.

## Data Availability

The data used in this study are available in the public repositories listed in Table 1. Specifically, the NCBI BioProject accession numbers are PRJNA693659, PRJNA693663, PRJNA983278, and the GSA accession code is PRJCA008752. The source code employed for data processing and analysis is available at the following GitHub repository: https://github.com/llosadac/Sjogren-s-syndrome-Microbiome-Analysis.
